# Initial Assessment of the LEO Based Navigation Signal Augmentation System from Luojia-1A Satellite

**DOI:** 10.3390/s18113919

**Published:** 2018-11-14

**Authors:** Lei Wang, Ruizhi Chen, Deren Li, Guo Zhang, Xin Shen, Baoguo Yu, Cailun Wu, Song Xie, Peng Zhang, Ming Li, Yuanjin Pan

**Affiliations:** 1 State Key Laboratory of Information Engineering in Surveying, Mapping and Remote Sensing, Wuhan University, Wuhan 430079, China; lei.wang@whu.edu.cn (L.W.); drli@whu.edu.cn (D.L.); guozhang@whu.edu.cn (G.Z.); xinshen@whu.edu.cn (X.S.); fenix@whu.edu.cn (P.Z.); lisouming@whu.edu.cn (M.L.); yjpan@whu.edu.cn (Y.P.); 2 Collaborative Innovation Center for Geospatial Technology, Wuhan, 430079, China; 3 State Key Laboratory of Satellite Navigation System and Equipment Technology, Shijiazhuang 050000, China; cetc_yubg@126.com (B.Y.); wucl54@126.com (C.W.); xies54@126.com (S.X.)

**Keywords:** Luojia-1A satellite, satellite-based augmentation system, GNSS signal augmentation, LEO navigation

## Abstract

A low Earth orbiter (LEO)-based navigation signal augmentation system is considered as a complementary of current global navigation satellite systems (GNSS), which can accelerate precise positioning convergence, strengthen the signal power, and improve signal quality. Wuhan University is dedicated to LEO-based navigation signal augmentation research and launched one scientific experimental satellite named Luojia-1A. The satellite is capable of broadcasting dual-frequency band ranging signals over China. The initial performance of the Luojia-1A satellite navigation augmentation system is assessed in this study. The ground tests indicate that the phase noise of the oscillator is sufficiently low to support the intended applications. The field ranging tests achieve 2.6 m and 0.013 m ranging precision for the pseudorange and carrier phase measurements, respectively. The in-orbit test shows that the internal precision of the ephemeris is approximate 0.1 m and the clock stability is 3 × 10^−10^. The pseudorange and carrier phase measurement noise evaluated from the geometry-free combination is about 3.3 m and 1.8 cm. Overall, the Luojia-1A navigation augmentation system is capable of providing useable LEO navigation augmentation signals with the empirical user equivalent ranging error (UERE) no worse than 3.6 m, which can be integrated with existing GNSS to improve the real-time navigation performance.

## 1. Introduction

The Global Positioning System (GPS) has brought a revolutionary way of providing high-accuracy spatial information. It has been successfully applied in many civil and military applications, which brings inestimable benefit. With recognizing the importance of global navigation satellite systems (GNSSs), more GNSS constellations are designed and deployed. In addition to the U.S. GPS system, the Russian GLONASS, the Chinese Beidou system (BDS), and the European Galileo system have also started providing full or initial positioning services. In addition, there are a few emerging regional navigation satellite systems, such as the Indian NavIC and the Japanese QZSS system. Multi-GNSS constellations bring more visible satellites, lower dilution of precision (DOP), and more reliable positioning solutions [1,2]. However, current GNSS constellations still face a number of challenges, such as the slow convergence in long baseline real-time kinematic positioning (RTK) and precise point positioning (PPP), signal blocking, and severe multipath effect due to city canyon, jamming, and spoofing, etc.

With regard to precise positioning applications, the differential positioning techniques, such as the RTK, the network RTK are limited by their service coverage. The non-differential approach, such as the PPP technique, requires 30–40 min to converge to centimeter-level accuracy, which hinders its applications [3]. In order to reduce the convergence time, the PPP ambiguity resolution technique was developed [4,5,6] which can solve the rapid re-convergence problem well, but has limited effect on the first convergence [7,8,9]. Another issue for the ambiguity fixed PPP technique is the reliability risk. There is no way to guarantee that the PPP ambiguities can be fixed 100 percent correctly.

With regard to the spoofing and jamming issues, there are also a few examples. A Chinese team named Unicorn demonstrated a GPS fraud case with only a simple record and playback device on DefCon 2015 hacker conference [10]. GPS signals are also reported to suffer from inference in Syria earlier in 2018 [11]. These problems are difficult to solve with one particular navigation technique. Therefore, next-generation positioning, navigation, and timing (PNT) systems require diverse techniques, including orbit diversity, non-satellite based navigation techniques, etc. [12,13]. Current GNSS constellations need to be augmented to extend their performance.

Generally, there are two types of satellite-based augmentation systems (SBAS): information augmentation and signal augmentation. For these information augmentation systems, the satellites act as the transponder, which retransmits the correction information uploaded from the ground facilities to users. The information augmentation can be achieved by the standard communication satellites, such as the Inmarsat, which have a wider bandwidth for transmitting more information than GNSS broadcast ephemeris [14,15]. The correction information may include the orbit correction, clock correction, ionosphere correction, integrity information, etc. [16,17]. The key to the information augmentation system is more focused on how to generate these corrections with the ground tracking stations. Signal augmentation means transmitting ranging signals by the satellite, so they can work together with current GNSSs, or even work stand alone. In order to generate high-quality ranging signals, high-quality oscillator and onboard ranging signal generation circuits are required for the signal augmentation implementation, so the signal augmentation requires specially designed payloads. The U.S. wide area augmentation system (WAAS) is a successful example of augmentation system based on geo-synchronized Earth orbit (GEO) platform, which combined information augmentation and signal augmentation. The Japanese QZSS also designed the LEX (L band experimental) signal for information augmentation. The Beidou system also broadcasts its own zonal correction via GEO satellite [18]. Generally, the information augmentation helps to improve positioning performance of current navigation signals in terms of precision or integrity. The signal augmentation extends the performance of current navigation constellation, in terms of availability, robustness, convergence, etc.

The navigation signals augmentation from LEO platform is expected to avoid the limitation of current GNSS systems and enhance the positioning and navigation performance. The early research exploring the benefit of LEO satellite to improve RTK performance is found in Trimble’s patent published in 1995 [19]. After that, there are a few pieces of research investigating the benefit of combining the Iridium constellation and current GNSS in terms of increasing visible satellites, reducing the positioning dilution of precision (PDOP) [20,21]. The integrity benefit from a dense LEO constellation also attracted research interests [22,23]. With fast-moving LEO platforms, the convergence time can be greatly reduced. Ge et al. reported that combining GPS/BDS/Iridium constellations can reduce the PPP convergence time from about 30 min to about 5 min [24]. Reid et al. explored the benefit of LEO constellation on Arctic aviation applications [25], which is also considered as a key technology for the marine navigation [26]. They also analyzed the error sources for LEO navigation signals and attempted to model them [27]. A few researchers even investigated the optimal design of broadcast ephemeris for LEO navigation satellites [28,29,30]. Rabinowitz systematically investigated certain challenging issues of LEO navigation receiver implementation [31]. In recent years, there have been a few ambitious commercial broadband LEO constellation proposals that can potentially serve as promising navigation sources. In such cases, the performance of the satellite positioning can be further improved [27]. Another benefit of LEO signal augmentation is that it is capable of transmitting stronger signals, which can even penetrate the building wall. With the orbit height reduced from 20,200 km to 650 km, the free space loss of the signal strength for 1.5 GHz signal may reduce up to 30 dB. The Iridium constellation announced their Satelles Time and Location (STL) service in 2016, which has successfully demonstrated the potential of indoor positioning applications and time synchronization applications with LEO navigation systems [32].

With recognizing the importance of LEO navigation augmentation system, the commercial aerospace industries also present great interests in developing LEO navigation augmentation systems as their value-added services. Researchers from Wuhan University are developing their space-based real-time information service system, which integrates the PNT service, remote sensing service, and communication service into a single satellite platform, which is named the ‘PNTRC’ concept [33,34]. The scientific experimental satellite Luojia-1A was successfully launched on 2 June 2018, carrying the integrated payload of night light photogrammetry and LEO navigation augmentation systems. They collected first-hand real LEO-based navigation augmentation signals from the Luojia-1A satellite for data analysis. In this research, the initial performance of LEO-based navigation augmentation systems from the Luojia-1A satellite is assessed.

## 2. The Principle of a LEO Navigation Augmentation System

**Figure 1:**
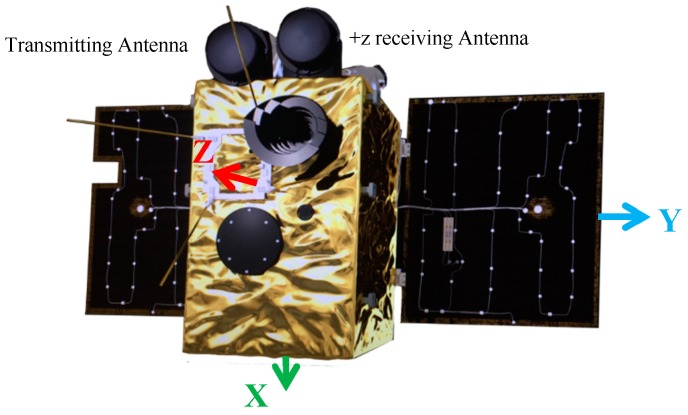
The physical configuration of the Luojia-1A satellite (+z side).

The Luojia-1A satellite is a lightweight scientific LEO designed by Wuhan University, intended for the experiment of signal augmentation based on a LEO platform. It has two major functions: the night light remote sensing and LEO signal navigation augmentation experiment. The satellite was launched on 2 June 2018 from the Jiuquan Satellite Launch Center, China. Luojia-1A employs a sun-synchronized orbit with 645 km orbit height with a local time of the descending node at 10:30 p.m. The external configuration of Luojia-1A is as shown in Figure 1. The satellite is about 19.8 kg and the envelop size is 870 mm × 520 mm × 390 mm with solar panel unfolded. The physical configuration of the satellite put strong constraints on the weight, size and power consumption of the navigation augmentation payload. The satellite equipped with three L band microstrip antennas on the satellite. Two of them are used to receive the GPS/Beidou signals and one for broadcasting the navigation augmentation signals. Distinguished from the ground-based signal augmentation system, such as the Locata system [35,36,37], the Luojia-1A satellite installed two antennas on +z and −z sides, respectively, to receive the GPS/Beidou dual-frequency signals. The satellite is tri-axis stabilized and the solar panels (−z side) face to the sun in standby mode. In this mode, −z side of the satellite faces to the outer space when the satellite locates between earth and sun. When the satellite moves to the other side of the earth, −z side faces toward the Earth and the +z antenna faces to the outer space. The two receiving antennas work simultaneously so that the Luojia-1A satellite is also capable of tracking the GNSS signals from the opposite side of the Earth. A similar GNSS antenna configuration can be found on COSMIC satellites [38,39]. The GNSS signals from the opposite antenna pair tracked GNSS signals independently and combined processing for orbit determination. The augmentation signal transmitting antenna is installed on the +z side of the satellite. During the transmission period, the +z side faces to the Earth. The diameter of the GNSS antennas is less than 10 cm and no choke rings are equipped on the antennas to reduce their weight.

Luojia-1A satellite is capable of transmitting dual-frequency band ranging signals, which are denoted as H1 and H2 in this study. There are very few publications documenting the signal augmentation techniques or results so far. As a low-cost experimental satellite, the principle of the navigation augmentation system is different from those navigation satellites, such as the Beidou or GPS. For the navigation satellites, the coordinate system, the time system and the quality of the ranging signals are particularly critical for the positioning/timing function. In the following section, the difference between the Luojia-1A satellite and current navigation satellites are discussed from these three aspects.

At first, the orbits of all the navigation satellites are observed by the ground monitoring stations. The broadcast ephemeris in the form of orbital elements are generated using the tracking data from the ground monitoring stations and then uploaded to the satellites [40]. Establishing and maintaining the coordinate framework for the navigation satellite system requires a geographically well-distributed tracking network, which can be costly and difficult to realize on a non-solid surface or inaccessible locations [41]. The onboard computation unit on the Luojia-1A satellite can automatically determine its orbit from received GPS/BDS dual frequency signals as if moving a ground station into space. From this prospect, Luojia-1A is a user of the current GNSS systems. The characteristic of the Luojia-1A satellite can be described by the orbit dynamics, so the precision of the geometrical orbit can be improved by incorporating the satellite dynamic models. With the dynamic smoothed algorithm, the satellite is capable of providing sub-meter level accuracy orbit in real-time, which achieves comparable or even better accuracy than the orbit in the GNSS broadcast ephemeris. With properly handled orbital and clock biases, the real-time onboard LEO orbit determination with the broadcast ephemeris reaches 0.3–0.4 m precision [42]. By autonomous orbit determination, Luojia-1A can maintain its coordinates in Beidou or GPS coordinate systems without setting up its own ground stations as if moving a Beidou or GPS satellite down to a lower orbit.

The heart of the navigation satellite is the high-performance atomic clock, which is the bottleneck of the navigation satellite performance and lifetime. Currently, most navigation satellites are equipped with 2~4 rubidium or cesium atomic clocks. The BDS3 and Galileo satellites even carry high-performance active hydrogen masers [43]. The one-second frequency stability of current GNSS clocks is around 10^−12^ [44]. The daily stability of the Beidou satellite reaches 10^−13^~10^−14^ [45,46]. These high-performance atomic clocks are often very large, heavy, and expensive, so they are not suitable for most micro/nano-satellite platforms. An alternative solution for the navigation system is equipping atomic clocks on the ground facilities rather than the space segment, such as the Chinese Area Positioning System (CAPS), but this method is only suitable for these GEO and inclined geo-synchronized orbit (IGSO) satellites [47,48]. Due to cost/size/weight constraints, the Luojia-1A satellite has to employ an oven-controlled oscillator (OCXO) rather than the high-performance atomic clock. The temperature control system of the satellite platform is used to ensure the temperature condition of the onboard OCXO. The short-term stable oscillator generates low phase noise, short-term stable impulses in order to maintain a sufficiently high-quality carrier phase generation. The oscillator is disciplined by the GPS/Beidou signals in-orbit, which calibrates the long-term frequency drift and maintains the frequency accuracy. With the in-orbit discipline technique, the time system of the Luojia-1A satellite is aligned to the GPS time or Beidou time at the level of tens of nanoseconds, which is satisfactory from the performance and cost perspectives.

With the orbit and the clock obtained, Luojia-1A is capable of generating its own ranging signals, which is driven by the onboard OCXO. The orbit and clock information is encoded and modulated on the carrier phase, and then broadcasted via the transmitting antenna. Driven by the same OCXO, the clock information determined by the receiver can be delivered to users via the transmitted signals. The hardware delay caused by the circuit is modeled as a constant bias. The phase noise is controlled within a low level by the low phase noise oscillator, which avoids phase lock loop fluctuation in the ground receiver.

As a satellite-based augmentation system, the interoperability and compatibility of current GNSS systems is an important issue. Although the Luojia-1A satellite is a scientific experiment satellite., we still rigorously evaluated its potential impact on other domestic and international satellite operators and we also coordinated with the satellite operators and potential ground users before satellite launch. We strictly controlled its emission power, spurious emission, experimental location, and duration to avoid unnecessary interference to other systems. Particularly, the Luojia-1A ranging signals are designed compatible with current GNSS signals and have no negative interference on current GNSS users. A strict electromagnetic compatibility (EMC) test on the payload was performed in an anechoic chamber before satellite assembly.

The Luojia-1A ground receiver is also developed, which is capable of tracking the GPS/Beidou and Luojia-1A augmentation signals simultaneously. With multiple visible LEO navigation augmentation satellite, users can benefit from the LEO signals and improve their positioning performance.

## 3. Ground Assessment of the Navigation Augmentation Signals

Before satellite assembly, the navigation augmentation payload was tested on the ground to ensure its performance. Many different tests have been undertaken, while the most important is the quality of the oscillator. The short-term stability of the oscillator can be evaluated in time-domain by Allan variance/Hadamard variance [49,50], or in the frequency domain by the phase noise [51].

**Figure 2:**
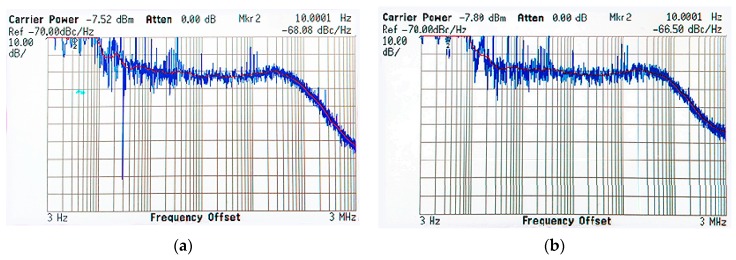
Ground assessment of Luojia-1A H1 (**a**) and H2 (**b**) signal phase noise.

The carrier phase noise is one of the most important frequency quality indicators, which directly impact the quality of the augmentation carrier phase signals. The phase noise is defined as a quality criterion for all navigation systems in their interface control documents. GPS/GLONASS requires the phase noise at f0 + 10 Hz no greater than 0.1 radians RMS [52,53] and Galileo is no greater than 0.04 radians RMS [54]. Beidou and QZSS defined a more detailed phase noise requirement at a different frequency offset [55,56]. The ground augmentation system Locata follows the GPS standard, but it works at 2.4 GHz [57]. The phase noise measured with the phase noise analyzer for the dual frequency band signals are presented in Figure 2. The blue lines are measured phase noise and the red lines are the smoothed phase noise. The figure indicates that the Luojia-1A satellite achieves excellent phase noise performance although it employs a miniature, low-cost OCXO. Luojia-1A satellite achieves better than −66 dBc/Hz at f0 + 10 Hz and better than 88 dBc/Hz at f0 + 1 kHz, which means the short-term stability of Luojia-1A signals is comparable with the QZSS and Beidou navigation satellites.

**Figure 3:**
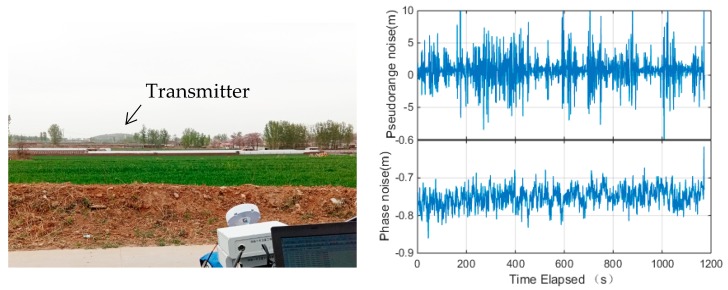
Ground assessment of the Luojia-A ranging measurements: the test scenario (**left panel**) and measured signal samples (**right panel**).

In addition to the laboratory test, the measurement precision was assessed by the field test. The payload was set up on the top of a small hill and the receiver was set up at about 2.5 km away from the transmitter. There was no tall building obstruction in the line-of-sight direction between the receiver and the transmitter, but there were a few trees and low buildings. The setup of the experiment is shown in the left panel of Figure 3. A signal attenuator is applied at the transmitter side to control the transmitting power.

A chip-scale atomic clock (CSAC) is equipped at the receiver side to mitigate the receiver clock effect. The nominal one-second frequency stability of the CSAC is better than 1.5 × 10^−10^. Applying a high-quality oscillator at both the transmitter and receiver side is to ensure the clock frequencies are stable over the experiment period. For the ground ranging signals, the atmosphere delay is negligible, so the ranging observations can be expressed as:

(1) $$\begin{array}{l} P_{i}=\rho +c(\delta t^{S}-\delta t^{R})+\epsilon_{Pi}\\ L_{i}=\rho +c(\delta t^{S}-\delta t^{R})+\lambda_{i}N_{i}+\epsilon_{\phi i} \end{array}$$

where $P_{i}$ and $L_{i}$ are the pseudorange and carrier phase measurements on the *i*th frequency expressed in meters. ρ is the geometrical distances. $\delta t^{S}$ and $\delta t^{R}$ are the satellite clock bias and the receiver clock bias, respectively. $\lambda_{i}$ and $N_{i}$ are the wavelength and unknown integer cycle number of carrier phase (ambiguity) on *i*th frequency, respectively.$\epsilon_{Pi}$ and $\epsilon_{\phi i}$ are the measurement noise of the pseudorange measurements and carrier phase measurements, respectively.

For the static test, the geometry distance ρ does not change over time. The ambiguity can also be considered as a constant if no cycle slip occurs. The oscillators equipped in the transmitter and the receiver side are all high stable clocks, so the clock biases can be modeled as constants or slopes over the time span. In this case, the measurement noise for the ground test can be evaluated by the time differenced approach. Assuming the temporal correlation between the measurements is negligible, the measurement noise can be expressed as:

(2) $$\begin{array}{l} \epsilon_{Pi}=\frac{1}{2}\Delta P_{i}-c(\Delta \delta t^{S}-\Delta \delta t^{R})\\ \epsilon_{\phi i}=\frac{1}{2}\Delta L_{i}-c(\Delta \delta t^{S}-\Delta \delta t^{R}) \end{array}$$

where Δ is the time difference operator. $\Delta \delta t^{S}$ and $\Delta \delta t^{R}$ are the differentiated clock biases, which correspond the frequency offset of the transmitter and the receiver. If the oscillator frequency is stable enough, the estimated measurements should be around a constant. If the oscillators between the transmitter and the receiver are perfectly synchronized, the differentiated clock bias $\Delta \delta t^{S}$ and $\Delta \delta t^{R}$ should be zero.

The measurement noise of the navigation augmentation system is presented in the right panel of Figure 3. The figure indicates that the pseudorange measurements of the navigation augmentation system are stable over time, which is benefit from the high stable oscillators. The constant bias on the carrier phase is caused by the inaccurate receiver or transmitter frequency. Assuming the autocorrelation in the pseudorange and carrier phase is negligible, the standard deviation of the pseudorange and carrier phase measurements is 2.66 m and 0.013 m, respectively. The pseudorange measurement precision is comparable with current GNSS signals, while the carrier phase noise is slightly larger than GNSS signals but still promising. Due to the experiment employs direct ranging measurement, the clock jitter at the transmitter and the receiver may affect the carrier phase measurement accuracy. Moreover, the signals are transmitted horizontally along the ground, so the roughness of the ground will reflect the signals and introduce complex multipath effect for the ground test. Overall, the ground test verified that the OCXO equipped on Luojia-1A performs well enough for the precise ranging signal generation.

## 4. In-Orbit Performance Evaluation Results

The Luojia-1A satellite was launched into space on 2 June 2018. Then, the in-orbit performance evaluation was carried out for one month and Wuhan University started freely distributing the night light image data from Luojia-1A satellite from 9 July. During this period, the LEO navigation augmentation system is evaluated from the three aspects: the orbit, the clock, and the signal quality.

### 4.1. In-Orbit Evaluation of the Orbit and Clock

Luojia-1A satellite maintains the time and coordinate system via onboard orbit determination procedure rather than the ground test facilities. The accuracy of the time/coordinate system maintenance would affect the augmentation performance. Hence, the precision of onboard orbit and clock determination should be evaluated.

**Figure 4:**
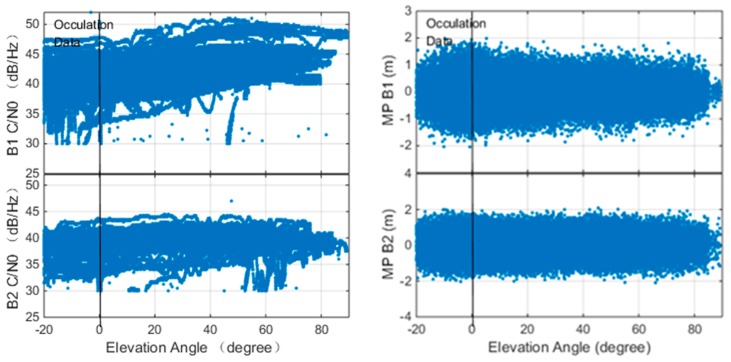
C/N0 (**Left**) and multipath (**Right**) of Luojia-1A onboard Beidou B1/B2 observation.

The precision of the onboard orbit and clock is affected by the quality of the onboard GNSS observation quality and the algorithm implementation. In this study, the Beidou observation quality of onboard receiver is assessed and the results are presented in Figure 4.

The left panel of Figure 4 shows the relationship between the carrier to noise ratio (C/N0) of the B1/B2 signals and the elevation angle. The data is collected with a 1 Hz sampling rate. The figure indicates that the maximum C/N0 of B1 and B2 are about 50 dB·Hz and 46 dB·Hz, respectively. The C/N0 of B2 signals is slightly lower than B1 since the antenna gain for B2 frequency is not as good as B1. The figure indicates that the C/N0 is higher for the high elevation angle case. A remarkable difference between the LEO observations and the ground observations is that LEO receivers also capable of tracking the observations with negative elevation angle. Luojia-1A is equipped with two receiving antennas facing the opposite direction, so it is capable of tracking observations up to −20°. The LEOs equipped with single GNSS antennas are also capable of tracking signals up to −5°, such as the CHAMP mission [58]. The observation with negative elevation angle is received from the other side of the Earth. The signals penetrated the atmosphere of the Earth and bent by the varying atmosphere reflectivity, which can be used to retrieve the ionosphere/troposphere profile [59,60].

The relationship between the multipath of B1 and B2 pseudorange and the elevation angle is presented in the right panel of Figure 4. The pseudorange and the carrier phase measurements from Luojia-1A satellite onboard receiver can be briefly expressed as:

(3) $$\begin{array}{l} P_{i}=\rho +\delta_{orb}+c(\delta t^{S}-\delta t^{R})+I_{i}+\delta_{trop}+\epsilon_{Pi}\\ L_{i}=\rho +\delta_{orb}+c(\delta t^{S}-\delta t^{R})-I_{i}+\delta_{trop}+\lambda_{i}N_{i}+\epsilon_{\phi i} \end{array}$$

where $\delta_{orb}$ is the orbital error. $I_{i}$ and $\delta_{trop}$ are the ionospheric delay and tropospheric delay, respectively. The definition of the rest terms is the same as Equation (1)

The multipath value is computed from [61]:

(4) $$\begin{array}{l} MP_{1}=P_{1}-L_{1}+\frac{2}{\alpha -1}(L_{2}-L_{1})\\ MP_{2}=P_{2}-L_{2}+\frac{2\alpha }{\alpha -1}(L_{2}-L_{1}) \end{array}$$

where $MP_{1}$ and $MP_{2}$ are the multipath statistics, $P_{1},P_{2}$ and $L_{1},L_{2}$ are the pseudorange and carrier phase measurements expressed in meters. $\alpha =f_{1}^{2}/f_{2}^{2}$, where $f_{1}$ and $f_{2}$ are the frequency of *L*1 and *L*2. It is noticed that the multipath statistics includes the carrier phase bias and the pseudorange multipath. As long as the carrier phase ambiguity does not change, the standard deviation of $MP_{1}$ and $MP_{2}$ is the same as the pseudorange multipath.

The figure indicates that the pseudorange multipath is less significant for higher elevation angle cases. Since the GNSS signals are tracked by two GNSS antennas in opposite directions, The Luojia-1A satellite is capable of tracking signals from negative elevation angle satellites. Due to the obstruction of the Earth, the satellite is not able to track observations lower than −20°. Generally, the multipath error of B1 and B2 are less than two meters. The standard deviation of B1 and B2 are 0.31 m and 0.44 m, respectively. The B2 signal is noisier due to its low C/N0. Comparing the Beidou observations collected by Fengyun-3C [62], the Luojia-1A GNSS observations have lower pseudorange noise level.

**Figure 5:**
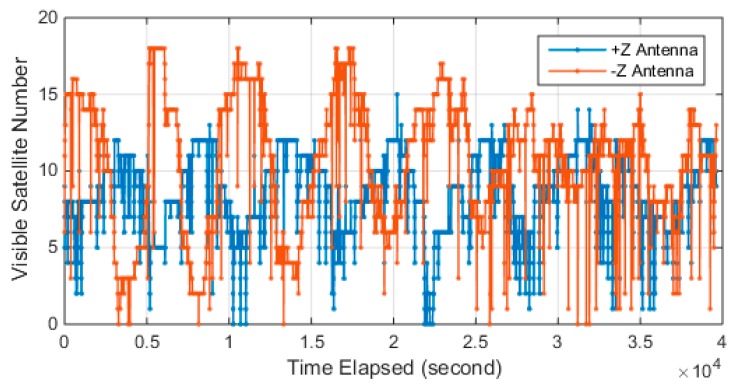
Visible satellite number of different +Z and –Z GNSS antennas.

Luojia-1A equipped two receiving antennas for GNSS signals retrieving since the solar panel of the satellite faces toward the sun in standby mode. The number of tracked GPS/Beidou is presented in Figure 5. The figure shows there is a significant period on the tracked satellite number and two antennas work in turn. The onboard receiver is capable of retrieving 12 GPS satellite and 12 Beidou satellites simultaneously. The maximum visible satellite number for −z antenna reaches 18, while +z tracked 15 satellites at maximum. The −z antenna tracked more visible satellites than the +z antenna since the Beidou satellites are not evenly deployed. The figure also indicates that there is some loss of lock events that happened on both the +z antenna and −z antenna, which still need to be investigated.

Considering the latency of onboard data processing, the real-time orbit is extrapolated for a short period and encoded into the broadcast ephemeris. The receivers decode the broadcast ephemeris and extrapolating the orbit to the time of concern. Due to the short visible window, the broadcast ephemeris design has different requirements as current GNSS satellites. There are a few studies discussing how to reasonably select the broadcast parameters to represent the centimeter-level LEO orbit [28,29,30]. However, all these methods are curve fitting based on precise orbit determination results. They are not suitable for onboard autonomous ephemeris generation. Instead of long-term fitting algorithms, Luojia-1A employs a rapid updating rate algorithm for the broadcast ephemeris. The orbit elements in the broadcast ephemeris are updated every 30 s, and the clock information is updated every 6 s. The clock information is updated more frequently to mitigate its extrapolation error. Considering the complexity of the ground observing condition, broadcast ephemeris redundancy scheme and parity scheme is adopted to facilitate the error correction during the ephemeris decoding procedure.

**Figure 6:**
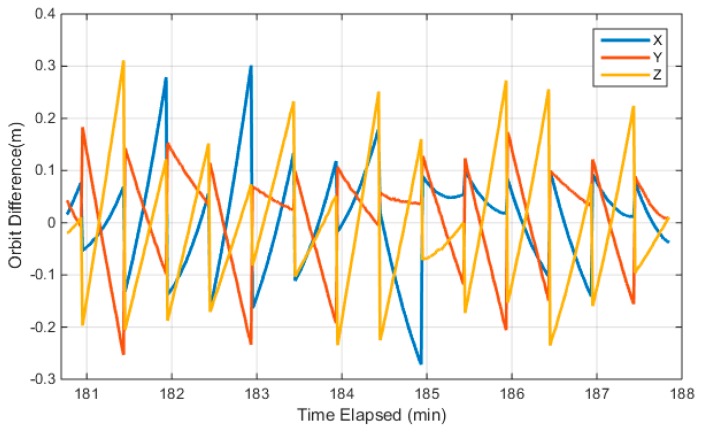
Internal precision of the Luojia-1A broadcast ephemeris.

Currently, Luojia-1A only broadcasts the navigation augmentation signal over China, so only a short-arc orbit is accessible from the broadcast ephemeris. The Luojia-1A satellite is not equipped with laser retroreflector arrays. Therefore, the external validation with the laser ranging technique is not feasible. Furthermore, the absolute orbit precision assessment is not feasible for the Luojia-1A satellite. In this study, the orbit calculated from the broadcast ephemeris is compared with the dynamic smoothed orbit. The dynamic smoothed orbit is a fusion solution of GNSS observations and satellite dynamic information with a Kalman smoothing algorithm in post-processing mode. Since only a part of the orbit is visible, the dynamic smoothed orbit subject to systematic bias with respect to its true position. As this bias can be assimilated into the clock term, the bias has limited adverse impact on positioning. The satellite orbit is extrapolated to 1Hz with the user algorithm of the Luojia-1A broadcast ephemeris and compared with the dynamically-smoothed orbit. The comparison results are presented in Figure 6. The figure indicates that the internal precision of the Luojia-1A broadcast ephemeris is 0.09 m, 0.08 m and 0.11 m for the X, Y, and Z direction in the Earth center Earth-fixed (ECEF) frame. The maximum discrepancy between the broadcast ephemeris and the dynamic smoothed orbit is about 0.3 m for 30 s extrapolation. The discontinuity of the orbit error is caused by the ephemeris update. The figure indicates that the user algorithm introduces decimeter level orbit error after 30 s extrapolation, which is considered acceptable. On the other hand, it also shows that 30 s updating interval is proper for the current user algorithm.

During the augmentation signal transmitting, the onboard OCXO is activated. The clock bias is determined from the onboard orbit determination and time synchronization method and then broadcasted to the users. The onboard GNSS receivers and the ranging signal transmitter is driven by the same OCXO, which ensures the precise time synchronization between the onboard receiver and transmitter. The latency caused by the onboard circuit is affected by the temperature. The temperature of the payload is controlled by the active temperature control system of the satellite platform. In this study, the hardware delay is modeled as a constant during the augmentation experiment. Considering the complexity of the space environment and the satellite motion status, the in-orbit oscillator behavior needs to be evaluated. Since the OCXO presents significant frequency drift, the stability of the clock is assessed with the Hardamard variance [49,50]. Given the phase data $\{x_{i}\},i=1,2,\cdots ,N$ with sampling interval $\tau_{0}$, then the Hardamard variance can be calculated with:

(5) $$\sigma_{H}^{2}(\tau )=\frac{1}{6\tau^{2}(N-3)}\sum_{i=1}^{N-3}{\left[x_{i+3}-3x_{i+2}+3x_{i+1}-x_{i}\right]}^{2}$$

where $x_{i}$ is the *i*th phase values at the averaging time τ.

**Figure 7:**
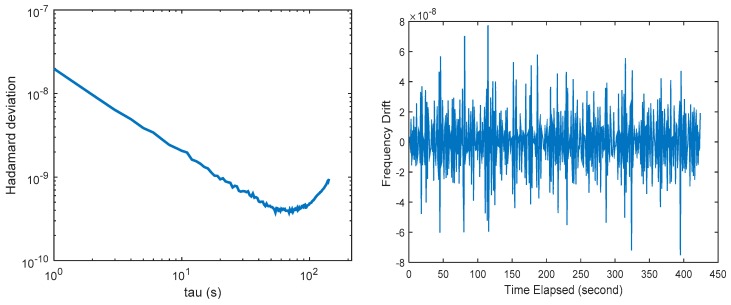
Hardamard variance of the satellite clock (**left panel**) and the frequency drift of the satellite clock (**right panel**).

The Luojia-1A satellite carries a small, low-cost OCXO as its frequency standard, whose nominal frequency stability is 1 × 10^−10^. The clock assessment results are presented in the left panel of Figure 7. The figure indicates that the clock equipped on Luojia-1A presents different characteristics with the navigation satellite clocks. The in-orbit stability of the oscillator reaches 3 × 10^−10^, which is achieved at τ=70 s. The frequency drift results in a quadratic form in the clock bias. Fortunately, the frequency drift does not affect the short-term clock stability and the signals tracking.

The right panel of Figure 7 presents the frequency drift observed by the onboard oscillator. The time series is rather noisy, with a non-zero mean. The average frequency drift for the oscillator is 2.68 × 10^−10^. Since the relativistic effect on the oscillator is not compensated in Luojia-1A, so the frequency drift is likely to be caused by the relativistic effect. The impact of the relativistic effect (sum of general and special relativistic) for Luojia-1A frequencies is around 2.5 × 10^−10^, so whether there is remaining frequency drift need to be further verified. The figure indicates that the time series is fairly stable, so the frequency drift can be treated as a constant, which can be calibrated in the data processing stage.

### 4.2. Navigation Augmentation Signal Noise Evaluation

**Figure 8:**
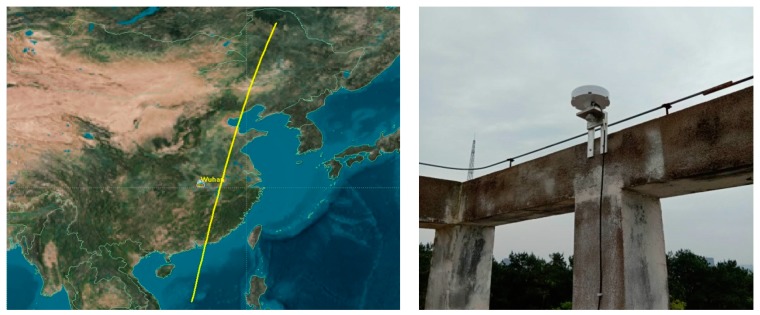
Luojia-1A satellite footprint (**left panel**) and the setup of the ground receivers on a rooftop structure (**right panel**).

The first navigation augmentation experiment of Luojia-1A is carried out on 9 June 2018 and the navigation augmentation signals from Luojia-1A were successfully tracked. The ground receiver of the Luojia-1A satellite is installed in Wuhan. The Luojia-1A receiver is capable of tracking GPS/Beidou dual-frequency observations and the Luojia-1A ranging signals. The Luojia-1A satellite ground track is presented in the left panel of Figure 8. Luojia-1A transmits ranging signals only in China to avoid potential signal inference to these international satellite operators. During the experiment period, the satellite moves toward the south direction. Although the GNSS antenna has been installed on the roof of the building, it is still obstructed by a roof ridge on the south direction.

**Figure 9:**
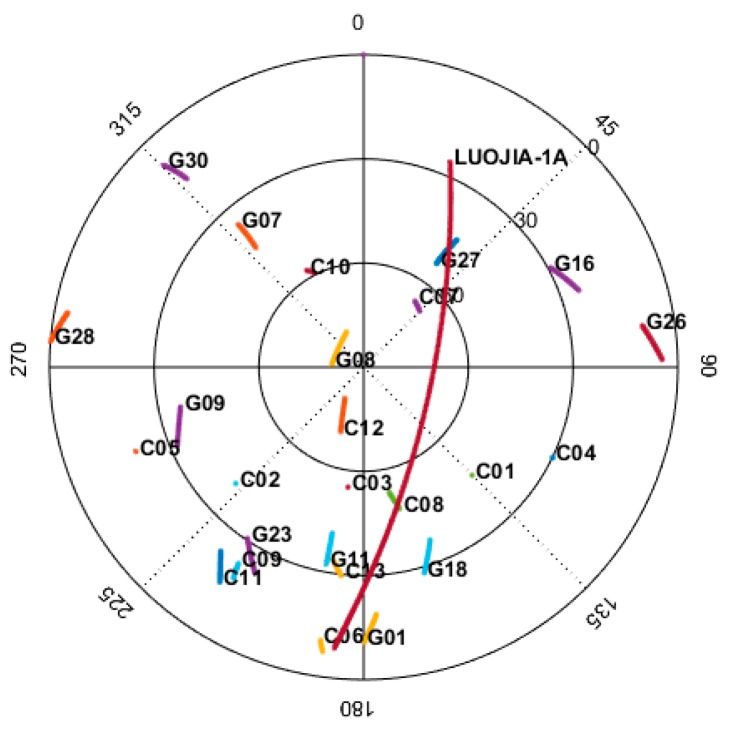
Representative sky plot of the Wuhan station shows the tracks of Luojia-1A and GPS/Beidou satellites over a 10 min pass.

One satellite pass lasts about 10 min and the sky plot during the experiment is presented in Figure 9. The figure indicates that the elevation angle of the slow-moving GPS and Beidou ascends or descends 9.24° at most, while the elevation angle of the Luojia-1A varies more than 107° over the 10 min. For this particular satellite pass, the maximum elevation angle for the Luojia-1A satellite reaches 70°. One of the most significant benefits of LEO navigation satellite is its fast-changing geometry, which helps to reduce the convergence time of PPP and long baseline RTK. For the Wuhan station, the Luojia-1A satellite has 4–5 visible passes per day and the total visible time is 30–40 min.

The tracked navigation augmentation signals from Luojia-1A satellite is used for the pseudorange noise analysis. Since there is only one LEO satellite in orbit, it is impossible to evaluate the receiver noise with the popular double differenced method. Therefore, the pseudorange noise is evaluated with the single receiver approach. The test statistics are constructed with:

(6) $$\begin{array}{cc} {\hat{\varepsilon }}_{P,i} & =(P_{i,t}-P_{i,t-1})-(L_{i,t}-L_{i,t-1})\\ & \approx 4\Delta I_{i}+2(1-\rho )\epsilon_{P} \end{array}$$

where ${\hat{\varepsilon }}_{P,i}$ is the test statistics for the pseudorange noise on *i*th frequency. $P_{i},L_{i}$ are the pseudorange, carrier phase measurement on *i*th frequency expressed in meters. The subscript t,t−1 refers the current epoch and previous epoch.$\Delta I_{i}$ refers to the time-differenced ionosphere delay. ρ refers to the autocorrelation between the pseudorange measurement. $\varepsilon_{P}$ refers to the pseudorange noise. In the equation, the carrier phase noise is ignored and no cycle slip is assumed. The ionosphere variation rate is normally less than 1 cm/s, so it can be neglected [63]. The pseudorange measurement is not filtered, hence the autocorrelation also can be neglected, and then the observed test statistics approximated equals to twice pseudorange noise.

**Figure 10:**
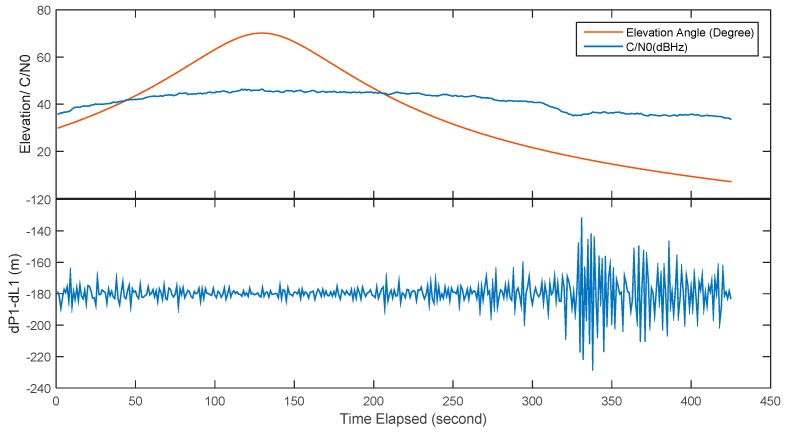
The pseudorange noise of Luojia-1A H1 signal.

The observed pseudorange noise for the frequency H1 is presented in Figure 10. The figure shows that the tracked satellite path length is about 7 min. The signals are locked when the satellite elevation is at 25° and lost lock at eight degrees. The elevation angle of acquisition and loss-of-lock is not the same since the acquisition sensitivity and the tracking sensitivity of the receiver are not the same. In addition, the signal tracking is a time-consuming procedure especially for these LEOs with large Doppler frequency shift. The figure shows that the signal is stably locked and no loss of lock occurs during the satellite pass. The C/N0 changes as the elevation angle change and the highest C/N0 is only 46.4 dB·Hz, which is significantly lower than the theoretical C/N0, suggesting that the radio frequency circuit and the antenna need to be further optimized. There is a sudden drop in the second half of the observation window, most likely to be is caused by signal obstruction since the sudden observation noise change also presents in subsequent experiments. The figure indicates that the overall standard deviation is 6.4 m. The observation noise generally dependent to the elevation angle, but the observation noise dramatically increases at low elevation case due to the signal obstructions and multipath effect. For the elevation higher than 20° case, the observation noise is reduced to 3.2 m. It is also noticed that there is a constant bias in the statistics, which is caused by the satellite clock drift. Since the code tracking loop of the receiver is not aided with the carrier phase information, it is vulnerable to the multipath effect. Considering the large acceleration in the line-of-sight direction between the satellite and the receiver, it is still a challenge to improve the code noise from the tracking loop design, but there is no doubt that the pseudorange precision can be improved significantly with better understand of LEO signal characteristics.

**Figure 11:**
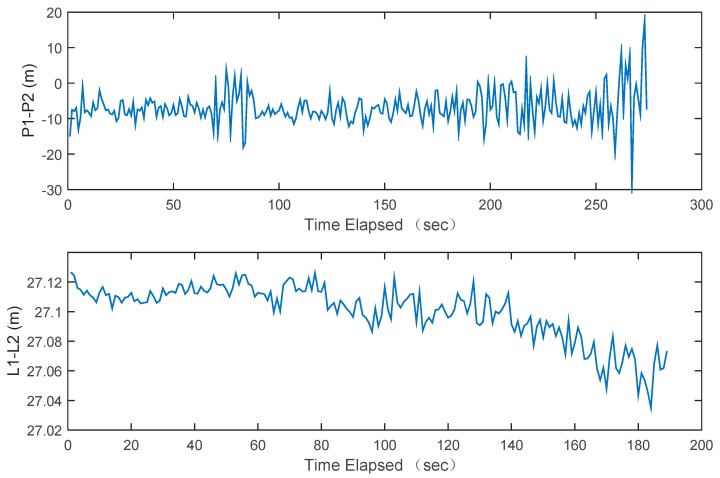
The observation noise structure of Luojia-1A pseudorange and carrier phase measurement evaluated via the geometry-free combination.

Luojia-1A is capable of transmitting dual frequency ranging signals simultaneously. The geometry-free (GF) combination of the pseudorange and carrier phase measurements is formed to evaluate the inter-frequency consistency. The geometry-free combination is defined as:

(7) $$\begin{array}{l} P_{GF}=P_{1}-P_{2}=I_{1}-I_{2}+2\varepsilon_{Pi}\\ L_{GF}=L_{1}-L_{2}=I_{1}-I_{2}+(\lambda_{1}N_{1}-\lambda_{2}N_{2})+2\varepsilon_{\phi i} \end{array}$$

where and are the geometry-free combination of the pseudorange and carrier phase measurements. The GF combination eliminated most errors except the ionosphere and the ambiguities. The ionosphere delay is also significantly mitigated and the ambiguities are constant over time. Therefore, the GF combination can be used to roughly evaluate the measurement noise. The GF combination of the pseudorange and the carrier phase measurements from Luojia-1A are presented in Figure 11. The standard deviation of the GF combination of the Luojia-1A pseudorange is 4.7 m. Assuming that *P*_1_ and *P*_2_ are independent, then the standard deviation of the pseudorange measurement is 3.3 m (1*σ*). The observation noise presents strong correlation as the elevation angle The GF combination of the carrier phase measurement is presented in the lower panel of Figure 11, which indicates that the standard deviation is 1.8 cm. Due to large acceleration in the line-of-sight direction (up to 77 m/s^2^) between the LEO and receiver, it is difficult to balance the sensitivity and the tracking loop precision. We still attempt to optimize the tracking loop parameters of our ground receiver to obtain better accuracy.

### 4.3. Handling Error Sources of the Ranging Measurements

There are a few LEO navigation constellations, such as the U.S. Transit system [64] and the Russian Parus system [65]. All of them are Doppler based positioning systems, so the error sources are simpler than the range based navigation system. On the other hand, the error sources for the LEO based ranging measurement are not exactly the same as the medium/geo-synchronized earth orbit navigation satellites. With the Luojia-1A pseudorange measurements and carrier phase measurements, their error sources are classified into three types: satellite specific, the propagation path specific and the receiver specific [66]. The satellite specific error includes the orbit error, the satellite clock error, the relativistic effect, the satellite hardware delay bias, the satellite antenna phase center offset (PCO), phase center variation (PCV), solar radiation error, albedo error, attitude error and phase windup error, etc. The propagation path specified error source includes the troposphere delay, the ionosphere delay, and the multipath error. The receiver specific error includes the receiving antenna PCO, PCV, the receiver clock bias and the receiver hardware biases.

The uniqueness of the LEO error sources characteristics lies in the following aspects:

1. The satellite hardware delay bias. Since the time system of the Luojia-1A satellite is aligned to the GPS time. The onboard GNSS receiver can only provide the time of receiving, rather than transmitting. The time consumption of baseband processing, position/velocity/timing computation, the ranging signal generation and the circuit delays all contribute to the hardware delays. Although some of the delays are compensated based on laboratory calibration, it still varies due to the complex space environment and temperature variation. Therefore, how to calibrate the hardware biases of the transmitter is challenging.
2. The relativistic effect. Due to low orbit height, the relativistic effect calculation method for the medium earth orbiters and GEOs is not directly applicable to the LEOs since the Earth cannot be treated as a mass point in this case. For the LEO, the J2 term of the gravity field has to be considered during the relativistic correction calculation [67,68]. The average term of relativistic effect is not compensated in the ground test, so a new relativistic model should be developed for LEO navigation augmentation satellites.
3. The satellite phase center offset. For the GNSS satellites, the PCO refers to the phase center offset with respect to the mass center. For Luojia-1A satellite, it refers to the offset between the −z receiving antenna and the transmitting antenna installed on the +z side. The offset for Luojia-1A is a few decimeters. The transmitting beam angle of Luojia-1A satellite is also not the same as the GNSS. For the MEOs, the beam width is 14.6° to cover the earth [69]. While the beam width has to be up to 60° for Luojia-1A satellite to achieves maximum service coverage. Hence the satellite PCV also has different characteristics.
4. The ionosphere delay of the ranging signals. Luojia-1A satellite is moving in the F2 layer of the ionosphere, while most GNSS satellites are located above the ionosphere. Hence the empirical ionosphere delay model based on GPS observations, such as the Klobuchar model may not work well for the Luojia-1A ranging signals.

Since there is only one such type LEO satellite in current operation, it is not possible to model all the errors considering some of them are not separable. For example, the satellite clock error and the receiver clock bias are not separable for only one satellite scenario, but this problem can be dealt with the datum transformation method. With properly defined clock datum, the receiver clock bias estimation is still possible. How to integration the Luojia-1A ranging signals and the legacy GNSS signals is requires substantial effort due to the presence of these biases. With more visible LEO satellites, once the receiver clock bias becomes estimable, then the LEO satellites would be treated as a new satellite system in data processing.

In order to validate the correctness of measurement, we calculate the measurement residuals by correcting the biases from the empirical models or external information. The observation model for Luojia-1A signals can be expressed as:

(8) $$\begin{array}{l} v_{P,i}=P_{i}-(\rho -dt_{s}-rel+dt_{r}+I_{i}+T)\\ v_{L,i}=\lambda_{i}\phi_{i}-\lambda_{i}N_{i}-(\rho -dt_{s}-rel+dt_{r}-I_{i}+T) \end{array}$$

where $P_{i},L_{i}$ are the pseudorange, carrier phase measurement on *i*th frequency expressed in meters. ρ is the geometry distance, $dt_{s}$, $dt_{r}$ are the satellite clock and receiver clock, respectively. $I_{i}$ denotes the ionospheric delay on *i*th frequency and T is the troposphere delay. *rel* refers to the relativistic effect, which is the sum of the general and the special relativistic effect. $\lambda_{i}$ is the wavelength of the *i*th frequency. All of these terms are expressed in meters. $\phi_{i}$ and $N_{i}$ are the carrier phase measurement and ambiguity on *i*th frequency expressed in cycles. $v_{P,i}$ and $v_{L,i}$ are the residuals of the pseudorange and carrier phase measurement. The PCO and PCV are omitted in the measurement noise analysis.

**Figure 12:**
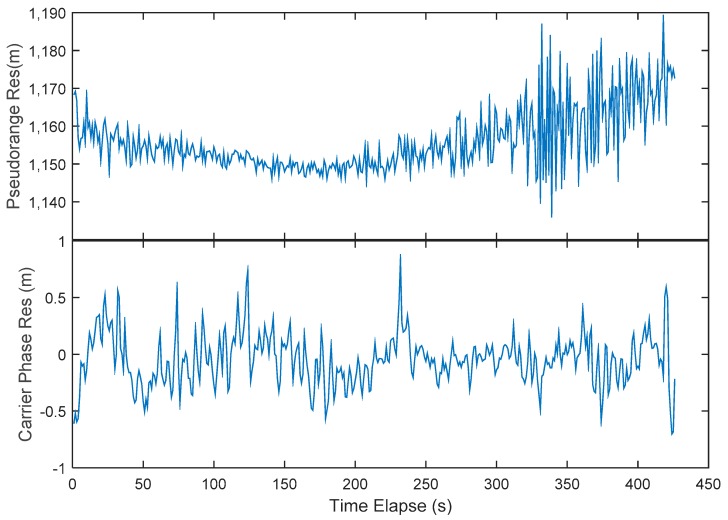
Representative pseudorange and carrier phase measurement residuals of the Luojia-1A with error sources corrected by empirical models.

In practice, the residuals are computed as follows: the geometry distance is computed from the known ground antenna position and the Luojia-1A broadcast ephemeris. The satellite clock is corrected according to the broadcast ephemeris as well. Due to the clock drift and the frequency drift, the satellite clock bias for the Luojia-1A reaches up to 1 μs, which corresponds several hundreds of meters, hence must be compensated. The general relativistic effect and special relativistic effect are corrected by the empirical model The total relativistic effect on GPS satellite is around $\Delta f=-4.5\times 10^{-10}\times f$, while for the Luojia-1A is about $\Delta f=-2.5\times 10^{-10}\times f$. The receiver clock is corrected by the clock bias estimated from GPS/Beidou observations. The magnitude of the receiver bias depends on the clock steering strategy. For the real-time clock steering, the receiver clock impact is at meter level. For the ground receiver used in the experiment, the clock bias is less than 30 m. The inter-system bias of the ground receiver is not derivable. Hence it is calibrated during the data processing. The Klobuchar model [70] and the Saastamoinen model [71] are used to correct the ionosphere and troposphere delay. Since there is no suitable ionosphere model for the LEO ranging signals, the Klobuchar model is adopted in the initial assessment stage. The ionospheric model for the LEO ranging signals should be refined in the future. The ionosphere delay and the troposphere delay are also elevation dependent errors, whose impact on the ranging signals is tens of meters at maximum. Then the satellite-specified hardware biases and the ambiguities are treated as a constant parameter and removed as well. For the positioning applications, these constant biases will be automatically assimilated into the receiver clock bias. As a result, they will not impact the positioning performance. After correction, the residuals of the pseudorange and the carrier phase measurement are presented in Figure 12. The standard deviation of the pseudorange residuals estimated with the raw measurements is 3.62 m, which includes the remaining orbit error, clock error, and the atmospheric errors. The figure indicates that the residuals are systematically biased at low elevation angle case, it is likely to be the remaining ionosphere residuals. The figure also shows there is a systematical constant offset, which is a combination of the hardware biases and inter-system bias. The precision of the residuals can be treated as the empirical user equivalent ranging error (UERE), which means that Luojia-1A satellite is capable of providing 3.62 m precision ranging signals in real-time for the navigation users. The carrier phase measurement achieves 0.22 m since standard temperature compensate oscillator (TCXO) with 0.05 ppm stability is used. With more precise receiver clock applied, e.g., the higher performing OCXO or chip-scale atomic clock (CSAC), the real carrier phase measurement accuracy can be assessed. There is no hardware biases presence in the carrier phase residuals since these biases have been calibrated along the ambiguities. The figure indicates that the carrier phase measurements are less noise than the pseudorange measurements, meaning that the adverse impact of multipath can be mitigated with the carrier phase smoothed technique for the LEO satellites. The figure also indicates that the pseudorange and the carrier phase measurements generated by Luojia-1A satellite are can be processed with current GNSS data processing theory. Therefore, they are suitable for navigation augmentation applications.

## 5. Conclusions and Future Work

This paper presents the initial results of the Luojia-1A satellite navigation augmentation system in-orbit validation. Wuhan University carried out the experiment of signal augmentation with the LEO platform. Luojia-1A satellite is capable of determining its own orbit and time standard in real-time and broadcasting its own dual frequency ranging signals. The ground assessment indicates that the phase noise of the payload is comparable with the GNSS signals, although a low-cost OCXO is employed. During the ground test, the precision of the pseudorange measurement and the carrier phase measurement achieves 2.6 m and 0.013 m, respectively. The in-orbit verification indicates that the observation noise of the onboard dual-frequency receiver is 0.31 m and 0.44 m for the Beidou B1 and B2 measurements. B2 has poorer measurement noise due to its lower C/N0. Equipped with two GNSS receiving antenna on the opposite direction, Luojia-1A satellite is capable of tracking GNSS signals down to −20° elevation, which has potential applications in GNSS occultation. The internal precision of the broadcast ephemeris is about 0.1 m, which does not include the systematical bias. The in-orbit stability of the clock reaches 3 × 10^−10^ and clock drift is observed during the in-orbit test. As a low-cost oscillator, its stability meets the expectation. The precision of the observed pseudorange measurement is assessed with the time differenced approach, and the precision of the navigation augmentation signals reaches 3.3 m for the H1 signal. With the dual-frequency measurement, the precision of the geometry-free combination of the pseudorange and carrier phase measurement reaches 1.8 cm. However, the pseudorange measurements suffer from severe multipath impact, which needs to be improved in the future. Finally, the correctness of the navigation augmentation is validated by calculating the residuals from the empirical models. After removing the error sources, the residuals of the augmentation signals are stable over a long period. After correcting the error sources with the empirical models, the precision of the pseudorange residuals is around 3.6 m. However, the remaining pseudorange residuals still present elevation-dependent biases, which need to be further studied. The precision of the carrier phase is limited by the clock phase noise of the ground receiver, which attained about 22 cm accuracy in the current stage. With a higher quality clock, centimeter-level accuracy carrier phase measurements can be expected. Generally, the assessment results verified that the methodology of the Luojia-1A satellite’s navigation augmentation system is practically feasible and the augmentation signals from Luojia-1A is useable. However, there is still a great deal of work to do in the future, including improving ground receiver performance, modeling the time synchronization errors, refining the error source models, and carrying out more experiments to investigate the benefit of combining GNSS/LEO for positioning and navigation applications. With better understanding of the LEO navigation augmentation signals, higher precision and better performance can be expected.
